# Accuracy of VO_2_ estimation according to the widely used Krakau formula for the prediction of cardiac output

**DOI:** 10.1007/s00059-023-05196-0

**Published:** 2023-07-13

**Authors:** Theresa Reiter, Julia Kerzner, Georg Fette, Stefan Frantz, Wolfram Voelker, Georg Ertl, Wolfgang Bauer, Caroline Morbach, Stefan Störk, Gülmisal Güder

**Affiliations:** 1 Department of Internal Medicine I, Cardiology Division, University Hospital Würzburg, Oberdürrbacherstr. 6, 97080 Würzburg, Germany; 2 Department of Clinical Research & Epidemiology, Comprehensive Heart Failure Center, University Hospital Würzburg, Am Schwarzen Berg 26, 97078 Würzburg, Germany; 3 Chair of Computer Science VI, University of Würzburg, 97074 Würzburg, Germany

**Keywords:** Cardiac output, $\dot {\mathrm{V}}$O_2_ approximation, Thermodilution, Indirect Fick method, Herzzeitvolumen, $\dot {\mathrm{V}}$O_2_-Approximation, Thermodilution, Indirekte Methode nach Fick

## Abstract

**Background:**

Invasive cardiac output (CO) is measured with the thermodilution (TD) or the indirect Fick method (iFM) in right heart catheterization (RHC). The iFM estimates CO using approximation formulas for oxygen consumption ($\dot {\mathrm{V}}$O_2_), but there are significant discrepancies (> 20%) between both methods. Although regularly applied, the formula proposed by Krakau has not been validated. We compared the CO discrepancies between the Krakau formula with the reference (TD) and three established formulas and investigated whether alterations assessed in cardiac magnetic resonance imaging (CMR) determined the extent of the deviations.

**Methods:**

This retrospective study included 188 patients aged 63 ± 14 years (30% women) receiving both CMR and RHC. The CO was measured with TD or with the iFM using the formulas by Krakau, LaFarge, Dehmer, and Bergstra for $\dot {\mathrm{V}}$O2 estimation (iFM-K/-L/-D/-B). Percentage errors were calculated as twice the standard deviation of the difference between two CO methods divided by their means; a cut-off of < 30% was regarded as acceptable*.* The iFM and TD-derived CO ratio was built, and deviations > 20% were counted. Logistic regression analyses were performed to identify determinants of a deviation of > 20%.

**Results:**

The TD-derived CO (5.5 ± 1.7 L/min) was significantly different from all iFM (K: 4.8 ± 1.6, L: 4.3 ± 1.6; D: 4.8 ± 1.5 L/min; B: 5.4 ± 1.8 L/min all *p* < 0.05). The iFM-K-CO differed from all methods (*p* < 0.001) except iFM‑D (*p* = 0.19). Percentage errors between TD-CO and iFM-K/-L/-D/-B were all beyond the acceptance limit (44/45/44/43%), while percentage errors between iFM‑K and other iFM were all < 16%. None of the parameters measured in CMR was predictive of a discrepancy of > 20% between both methods.

**Conclusion:**

The Krakau formula was comparable to other iFM in estimating CO levels, but none showed satisfactory agreement with the TD method. Improved derivation cohorts for $\dot {\mathrm{V}}$O_2_ estimation are needed that better reflect today’s patients undergoing RHC.

## Abstract (de)

**Hintergrund:**

Das Herzzeitvolumen (HZV) wird häufig mittels Thermodilution (TD) oder mit der indirekten Methode nach Fick (iFM) bei der Rechtsherzkatheterisierung (RHC) bestimmt. Bei der iFM wird das HZV anhand von Approximationsformeln für den Sauerstoffverbrauch ($\dot {\mathrm{V}}$O_2_) geschätzt, es bestehen allerdings signifikante Diskrepanzen zwischen beiden Methoden. Eine häufig angewendete aber bislang nicht validierte Formel ist die Formel nach Krakau. In der vorliegenden Arbeit wurden HZV-Diskrepanzen zwischen der Krakau-Formel und der Referenzmethode (TD) sowie mittels 3 etablierten Approximationsformeln verglichen. Zudem untersuchten wir, ob diese Unterschiede durch Parameter erklärt werden können, die in der kardialen Magnetresonanztomographie (CMR) gemessen wurden.

**Methoden:**

Insgesamt wurden 188 Patienten im Alter von 63 ± 14 Jahren (30% Frauen) eingeschlossen, bei denen sowohl eine CMR als auch eine RHC durchgeführt wurde. Das HZV wurde mittels TD oder mit der iFM bestimmt, wobei die Formeln von Krakau, LaFarge, Dehmer und Bergstra zur $\dot {\mathrm{V}}$O2-Schätzung (iFM-K/-L/-D/-B) verwendet wurden. Der prozentuale Fehler wurde berechnet als das Zweifache der Standardabweichung der Differenz zwischen beiden HZV-Bestimmungsmethoden, geteilt durch ihre Mittelwerte; ein Grenzwert von < 30 % wurde als akzeptabel angesehen. Der Quotient zwischen iFM-HZV und TD-HZV wurde gebildet, und die Abweichungen > 20 % ermittelt. Logistische Regressionsanalysen wurden durchgeführt, um Determinanten einer relevanten Abweichung von > 20 % zu erkennen.

**Ergebnisse:**

Das mittels TD gemessene HZV (5,5 ± 1,7 l/min) unterschied sich signifikant von sämtlichen mittels iFM geschätzten HZV-Werten (K: 4,8 ± 1,6; L: 4,3 ± 1,6; D: 4,8 ± 1,5 l/min; B: 5,4 ± 1,8 l/min; alle *p* < 0,05). Das HZV nach iFM‑K unterschied sich von allen anderen Methoden (*p* < 0,001) außer iFM‑D (*p* = 0,19). Die prozentualen Fehler zwischen TD-HZV und iFM-K/-L/-D/-B lagen alle außerhalb der Akzeptanzgrenze (44/45/44/43 %), während die prozentualen Fehler zwischen iFM‑K und anderen iFM alle < 16 % waren. Keiner der in der CMR erfassten Parameter, waren prädiktiv für eine Diskrepanz von > 20 % zwischen beiden Methoden.

**Schlussfolgerung:**

Bei der Schätzung des HZV war die Krakau-Formel vergleichbar mit anderen iFM, aber keine Formel ergab eine ausreichende Übereinstimmung mit der TD-Methode. Für die $\dot {\mathrm{V}}$O_2_-Schätzung sind modernere Ableitungskohorten erforderlich, die die heutigen Patienten, bei denen eine RHC durchgeführt wird, besser abbilden.

Invasive cardiac output (CO) assessment with the thermodilution (TD) or the Fick method during right heart catheterization (RHC) is a well-established examination applied to patients with various cardiac and pulmonary diseases [1, 2].

The TD method enables CO calculation by using the modified Stewart–Hamilton principle. A thermistor at the distal end of the Swan Ganz catheter measures the change in blood temperature in the pulmonary artery after saline injection of a definite volume and temperature in the proximal ending of the catheter [3, 4]. Although methodologically prone to errors that may influence the injectate alongside its path, as in higher-grade tricuspid valve insufficiency or severe heart failure, the TD method is generally regarded as the guideline-recommended practical “reference standard” for CO assessment and is only discouraged in patients with intracardiac shunt diseases [1, 5].

The direct Fick method is also recommended by the guidelines and is based on the principle that CO is equal to oxygen consumption ($\dot {\mathrm{V}}$O2), measured as the difference between inspired and expired oxygen content, divided by the difference in arterial and mixed venous oxygen concentration [1, 6]. For the direct measurement of $\dot {\mathrm{V}}$O_2_, tightly fitted facemasks are used that monitor the oxygen and carbon dioxide levels in the patient’s breath. Noteworthy is a significant variability in the measured $\dot {\mathrm{V}}$O_2_ levels, and therefore, repeat or continuous measurements are advocated [7]. Because the accurate measurement of $\dot {\mathrm{V}}$O_2_ in the catheter laboratory is demanding and time-consuming, the indirect Fick (iFM) method is preferably applied in clinical practice, since $\dot {\mathrm{V}}$O_2_ is estimated by different approximation formulas and not measured [2].

There are multiple formulas for $\dot {\mathrm{V}}$O_2_ estimation, with the equations proposed by LaFarge and Miettinen [8], Dehmer et al. [9], and Bergstra et al. [10] being the most frequently cited in the literature [11–13].

The correlation between TD and iFM-derived CO is much weaker than between TD and direct Fick-derived CO. In multiple studies, a CO discrepancy greater than 20% between both methods was reported, which is regarded as a clinically relevant threshold to indicate unsatisfactory agreement [14]. Deviations > 20% were described in up to 30–40% of cases [11, 15, 16]. It is unclear whether the discrepancies might be predisposed or enhanced by a compromised right ventricular function or other morphological constraints.

Besides these better-established formulas, Dr. Ingo Krakau introduced a $\dot {\mathrm{V}}$O_2_ approximate in his cardiac catheterization textbook, first published in 1999 [17]. This formula is used as the default setting for CO estimation by the iFM in many catheter laboratories, although its accuracy and performance in the clinical setting have not been validated to date. The present study aimed to compare CO, calculated as proposed by I. Krakau (iFM_Krakau_), with TD-derived CO and three established approximation formulas (i.e., iFM_LaFarge_, iFM_Dehmer_, iFM_Bergstra._). We further analyzed whether discrepancies between TD- and iFM_Krakau_-derived CO correlate with morphological or functional pathologies assessed in cardiac magnetic resonance imaging (CMR).

## Methods

### Study design and patient selection

In this retrospective, observational, single-center study, we evaluated the hemodynamic interrelations in patients undergoing RHC and CMR at the University Hospital of Würzburg. Patients were identified using the Data Warehouse of the University Hospital, a proprietary digital storage solution that connects and harmonizes all electronically stored patient data, including discharge letters, diagnosis coding schemes, laboratory values, and procedures such as echocardiography, CMR, cardiac catheterization, and others [18].

The study was conducted in compliance with the Declaration of Helsinki. Approval of the ethical committee was waived as the Data Warehouse runs on standard operating procedures that are controlled and approved by the institution’s data protection officer.

In total, 293 consecutive patients with information on RHC and CMR were identified between January 2016 and January 2022 via the Data Warehouse. Patients were excluded from the analyses if one of the following conditions applied: no valid concomitant CO measurement of either TD method or one iFM method (*N* = 69); invalid or insufficient CMR data (e.g., early termination for claustrophobia) or a time distance greater than 2 weeks between RHC and CMR (*N* = 26); shunt volume > 10% or significant congenital heart defects (*N* = 10). Accordingly, the current study refers to 188 patients*.* Based on electronic patient records, information was collected on cardiovascular diseases (CVD) and risk factors (CVRF), non-cardiovascular conditions, medication (with emphasis on the treatment of CVD and CVRF), electrocardiogram, echocardiography, coronary angiography, RHC, CMR, and laboratory parameters measured at the Central Lab of the University Hospital on the day of or the day before RHC.

### Transthoracic echocardiography

Transthoracic echocardiography was performed according to practice guidelines [19] as part of the clinical routine during or before the index hospitalization as part of an outpatient visit. In five patients, transesophageal echocardiography and not transthoracic echocardiography was available. The median time difference between echocardiography and RHC was 1 day (quartiles 0; 5 days).

### Cardiac magnetic resonance imaging

The CMR was performed on either a 1.5- or a 3.0‑T Achieva D scanner (Philips Healthcare, Best, The Netherlands) according to the Society for Cardiovascular Magnetic Resonance standard [20]. The median time difference between CMR and RHC was 3 days (quartiles 1, 6 days).

Data analysis was performed on the dedicated workstation IntelliSpacePortal (Philips Healthcare, Best, The Netherlands). Ventricular volumes were derived from a short-axis CINE stack covering the ventricles from the apex to the valvular plane. The endomyocardial border was traced manually for the end-systolic and end-diastolic phases, with the papillary muscle considered part of the intracavitary volume. The stroke volume (SV) was calculated as the difference between the end-diastolic (EDV) and the end-systolic (ESV) volume for both the left and the right heart. Ejection fraction (EF) was calculated from the division of SV and the EDV multiplied by 100.

### Right heart catheterization and formulas used

Depending on the indication, RHC was performed according to standard recommendations [21], either alone or in combination with coronary angiography, using the Vigilance II™ monitor (Edwards Lifesciences, Irvine, CA, USA) or the Schwarzer Cardiotek Evolution system (Schwarzer Cardiotek GmbH, Heilbronn, Germany). Hemoglobin and oxygen saturation of mixed venous blood (SVO_2_) were measured with the ABL80 FLEX CO-OX blood gas analyzer (Radiometer Medical ApS, Brønshøj, Denmark). Arterial oxygen saturation (SaO_2_) was either measured invasively if coronary angiography was additionally performed or derived from finger pulse oximetry. For body surface area (BSA), the formula from Dubois and Dubois was applied [21]. Data from RHC (hemodynamics and pressure tracings) were double-checked and entered manually by two cardiologists (TR and GG).

#### Thermodilution approach.

The TD was performed utilizing one of two True Size Thermodilution Swan-Ganz catheters (models 141F7 and 151F7; Edwards Lifesciences, Irvine, CA, USA). As an in-house standard, CO measurements were performed at least three times for patients with sinus rhythm. They were repeated until three similar measurements were obtained for patients with atrial fibrillation or with a discrepancy of > 10% from the mean [22, 23]. Only the mean of the TD measurements was available for the present analysis.

#### Indirect Fick approaches.

The CO was calculated as the ratio between estimated oxygen consumption ($\dot {\mathrm{V}}$O_2_) and the difference between arterial and mixed venous oxygen content using Eq. 1 [17]:

1 $$\mathrm{av}\mathrm{DO}_{2}=\text{hemoglobin}\,(\mathrm{g}/\mathrm{dL})*1.34\mathrm{\,ml}/\mathrm{g}*(\mathrm{a}\mathrm{S}_{\mathrm{O}2}-\mathrm{v}\mathrm{S}_{\mathrm{O}2}).$$

The four different formulas used for $\dot {\mathrm{V}}$O_2_ estimation are listed in Table 1.

**Table 1:** Equations used for $\dot {\mathrm{V}}$O2 estimation

Equation	$\dot {\mathrm{V}}$O2 (mL/min) in men	$\dot {\mathrm{V}}$O2 (mL/min) in women
Krakau [17]	$\mathrm{BSA}*(161-\mathrm{age}*0.54)$	$\mathrm{BSA}*(147.5-\mathrm{age}*0.47)$
LaFarge [8]	$\mathrm{BSA}*(138.1-11.49*\mathrm{Ln}(\mathrm{age})+0.378*\mathrm{HR})$	$\mathrm{BSA}*(138.1-17.04*\mathrm{Ln}(\mathrm{age})+0.378*\mathrm{HR})$
Dehmer [9]	$\mathrm{BSA}*125$	$\mathrm{BSA}*125$
Bergstra [10]	$\mathrm{BSA}*157.3+10-10.5*\mathrm{Ln}(\mathrm{age})+4.8$	$\mathrm{BSA}*157.3-10.5*\mathrm{Ln}(\mathrm{age})+4.8$

*BSA* body surface area, *Ln* natural logarithm, *HR* heart rate

### Statistical analysis

Data are described by count (percent), mean (standard deviation), or median (quartiles), as appropriate. Group comparisons were carried out for nominal and ordinal parameters using exact Fisher’s or chi-square tests and for metric parameters using Mann–Whitney *U* tests or the Kruskal–Wallis test. The TD-CO served as the reference standard and was compared with each method using the Wilcoxon signed-rank test and Pearson’s correlation coefficient (*r*). Bland–Altman plots illustrated the agreement between TD-derived CO and CO derived by different iFM methods [24].

The CO derived by iFM_*Krakau*_ was divided by TD-derived CO to obtain the percentage of patients deviating more than 20% from the reference standard. Patients’ characteristics were then compared in the three resulting groups with ratios < 0.8 versus 0.8 to 1.2 and > 1.2. The respective ratios were also computed for iFM_*LaFarge*_, iFM_*Dehmer*_, and iFM_*Bergstra*_. To quantify comparability, percentage errors were calculated as 1.96 times the standard deviation (SD) of the difference between TD-CO and iFM-CO divided by their means and expressed as percent (Eq. 2):

2 $$(1.96*\text{SD of TD-CO} - \text{iFM-CO}/(\text{TD-CO}+\text{iFM-CO})*0.5).$$

Percentage error estimates were also calculated between iFM_Krakau_-CO and other iFM-CO. As recommended by Critchley and Critchley, estimates of the percentage error < 30% were regarded as acceptable [14].

Predictors of a deviation > 20% from the reference TD-derived CO (ratio iFM_*Krakau*_-CO/TD-CO < 0.8 OR ratio iFM_*Krakau*_-CO/TD-CO > 1.2) were determined in univariable logistic regression analysis. Variables used for the index variable or their derivatives (e.g., TD-CO or SaO_2_) were excluded from the analysis. Significant univariate predictors (with *p* < 0.05) were included in a multivariable logistic regression analysis using the forward and backward selection methods. As the results were similar, only the backwards selection method is shown.

A significant group difference was assumed for all test procedures at a (two-sided) value of *p* < 0.05. All statistical analyses were performed using IBM SPSS Statistics for Windows Version 28.

## Results

### Study population and baseline characteristics

The mean age of the 188 patients was 63 (±14.4) years, and 30% were women. Their characteristics are shown in Table 2*. *In 10% of the cases, the indication for RHC was diagnosis or suspicion of pulmonary hypertension of non-cardiac origin (*N* = 18/188). The majority of RHC was performed for a cardiac reason: 78 patients had grade °III valvular heart disease (VHD; *N* = 28/78, 36% with accompanying HFrEF); 35 patients were suspected of having higher-grade VHD, which could be invasively excluded (*N* = 14/35; 40% with HFrEF); 54 patients had heart failure without higher-grade VHD (*N* = 11/54 had heart failure with preserved ejection fraction [HFpEF] of different origins); three patients were suspected of having constrictive pericarditis.

**Table 2:** Baseline characteristics

	*N*	All		iFM-CO-K/TD-CO					*p*< 0.8 vs. 0.8–1.2	*p*> 1.2 vs. 0.8–1.2
			*N*	< 0.8	*N*	0.8–1.2	*N*	> 1.2		
*Age, years*	188	63 (55; 75)	74	67 (57; 75)	103	63 (54; 75)	11	60 (52; 73)	0.26	0.75
*Men*	188	132 (70.2%)	74	47 (63.5%)	103	78 (75.7%)	11	7 (63.6%)	0.095	0.47
*Height, cm*	188	173 (165;179)	74	172 (164; 178)	103	175 (168; 180)	11	176 (164; 186)	**0.012**	0.67
*BMI, kg/m*^*2*^	188	26.2 (23.7; 30)	74	27.2 (24.2; 30)	103	25.5 (23.4; 29)	11	25.8 (23.1; 29)	0.14	0.75
*BSA, m*^*2*^	188	1.9 (1.8; 2.1)	74	1.9 (1.7; 2.1)	103	1.9 (1.8; 2.1)	11	2.1 (1.9; 2.2)	0.28	0.24
*Cardiac RHC indication*	188	170 (90.4%)	74	65 (87.8%)	103	94 (91.3%)	11	11 (100%)	0.46	0.60
HFrEF	188	84 (44.7%)	74	37 (50.0%)	103	41 (39.8%)	11	6 (54.5%)	0.22	0.36
VHD ≥ °3	188	78 (41.5%)	74	34 (45.9%)	103	41 (39.8%)	11	3 (27.3%)	0.44	0.53
*Comorbidities*										
CAD	188	82 (43.6%)	74	36 (48.6%)	103	39 (37.9%)	11	7 (63.6%)	0.17	0.12
Atrial fibrillation or flutter	188	65 (34.6%)	74	21 (28.4%)	103	40 (38.8%)	11	4 (36.4%)	0.20	1.0
*Medication*										
ACEi/ARB/ARNI	188	151 (80.3%)	74	62 (83.8%)	103	81 (78.6%)	11	8 (72.7%)	0.44	0.70
Beta-blocker	188	147 (78.6%)	74	58 (78.4%)	102	80 (78.4%)	11	9 (81.8%)	1.0	1.0
MRB	187	89 (47.6%)	74	36 (48.6%)	102	47 (46.1%)	11	6 (54.5%)	0.76	0.41
Loop diuretics	187	120 (64.2%)	74	56 (75.7%)	102	59 (57.8%)	11	5 (45.5%)	**0.016**	0.32
*Laboratory values*										
Creatinine, mg/dL	187	1.1 (0.88; 1.3)	73	1.0 (0.84; 1.2)	103	1.1 (0.91; 1.3)	11	1.1 (0.91; 1.8)	0.060	0.61
Hemoglobin, g/dL	187	13.7 (12.4; 15)	74	13.8 (12.6; 15)	103	13.7 (12.4; 15)	10	13.5 (12.2; 16)	0.89	0.73
*Echocardiography*										
LVEF, %	178	50 (30; 54)	71	45 (30; 53)	96	51 (31; 55)	11	40 (23; 53)	0.51	0.38
TAPSE, mm	179	18 (14; 22)	69	18 (15; 21)	99	18 (14; 22)	11	17 (12; 18)	0.97	0.18
TR-Pmax, mm Hg	169	29 (23; 43)	64	31 (24; 44)	95	29 (21; 38)	10	32 (20; 43)	0.14	0.84
AVS °III	188	21 (11.2%)	74	15 (20.3%)	103	5 (4.9%)	11	1 (9.1%)	*0.003*	0.46
MVR °III	188	25 (14.2%)	74	9 (12.2%)	103	14 (13.6%)	11	2 (18.2%)	0.83	0.65
TVR °III	188	14 (7.4%)	74	3 (4.1%)	103	11 (10.7%)	11	0 (0%)	0.16	0.31
*CMR*										
LVEF, %	188	46 (30; 59)	74	45 (30; 58)	103	48 (32; 59)	11	35 (24; 54)	0.34	0.18
LVEF ≤ 40%	188	78 (41.5%)	74	32 (43.2%)	103	39 (37.9%)	11	7 (63.6%)	0.54	0.12
LVEDD, mm	187	61 (54; 69)	73	62 (55; 70)	103	60 (53; 68)	11	63 (56; 69)	0.13	0.43
LA area, cm^2^	186	29 (23; 36)	72	29 (24; 36)	103	29 (22; 35)	11	27 (23; 35)	0.50	0.99
LVEDV, ml	187	195 (145; 276)	73	211 (161; 277)	103	189 (136; 276)	11	221 (158; 261)	0.16	0.69
LV-CO, L/min	187	5.8 (4.5; 7.0)	73	6.3 (4.7; 7.2)	103	5.6 (4.4; 6.8)	11	5.4 (4.9; 5.9)	0.24	0.75
LV SI, mL/m^2^	180	43 (35; 51)	70	44 (36; 51)	99	43 (36; 51)	11	35 (29; 41)	0.91	0.066
RVEF, %	186	53 (43; 62)	73	54 (42; 63)	102	54 (44; 62)	11	46 (39; 60)	0.82	0.23
RVEDD, mm	186	34 (29; 38)	72	35 (30; 38)	103	34 (29; 39)	11	30 (27; 35)	0.98	0.19
RA area, cm^2^	187	25 (20; 30)	73	25 (20; 30)	103	24 (19; 32)	11	25 (21; 30)	0.90	0.93
RVEDV, mL	186	158 (122; 195)	73	149 (120; 192)	102	162 (125; 206)	11	158 (136; 188)	0.13	0.53
RV-CO, L/min	186	5.4 (4.2; 6.8)	73	5.3 (4.3; 6.8)	102	5.6 (4.2; 7.3)	11	4.7 (4.6; 5.9)	0.60	0.34
RV SI, mL/m^2^	183	40 (33; 50)	73	39 (34; 47)	99	43 (34; 55)	11	33 (31; 39)	0.16	0.052
*RHC*										
mPAWP, mm Hg	186	13 (7; 20)	72	14 (7; 22)	103	12 (7; 18)	11	14 (10; 24)	0.26	0.15
mPAP, mm Hg	188	22 (16; 32)	74	25 (18; 34)	103	20 (15; 29)	11	21 (19; 31)	*0.007*	0.29
TD-CO, L/min	188	5.4 (4.4; 6.5)	74	5.9 (4.9; 6.8)	103	5.2 (4.3; 6.1)	11	4.1 (2.5; 6.4)	*0.005*	0.065
TD-CI, L/min/m^2^	188	2.8 (2.4; 3.3)	74	3.0 (2.6; 3.5)	103	2.6 (2.3; 3.1)	11	1.9 (1.4; 2.9)	*0.001*	*0.017*
TD-PVR, dyn*s/cm^5^	184	130 (93; 194)	72	145 (99; 197)	102	125 (82; 183)	10	171 (102; 270)	0.12	0.21

Unless indicated otherwise, values are presented as *N* (%) or median (25th–75th percentile). All *p* values refer to Fisher’s exact test, chi-square test, or the Mann–Whitney *U *test as appropriate; *p*≤ 0.05 values are marked in bold

*ACEi* angiotensin-converting enzyme inhibitor, *ARB* angiotensin receptor blocker, *ARNI* angiotensin receptor neprilysin Inhibitor, *AVS* aortic valve stenosis, *BMI* body mass index, *BSA* body surface area, *CAD* coronary artery disease, *CMR* cardiac magnetic resonance imaging, *HFrEF* heart failure with reduced ejection fraction, *LA* left atrium, *LVEDV* LV end-diastolic volume, *LV-CO* LV cardiac output, *LV-CI* LV cardiac index, *LVEF* LV ejection fraction, *LV SI* LV stroke index, *MRA* mineralocorticoid receptor blocker, *mPAWP* mean pulmonary artery wedge pressure, *mPAP* mean pulmonary artery pressure, *MVR* mitral valve regurgitation, *RA* right atrium, *RHC* right heart catheterization, *RV-CO* right ventricular cardiac output, *RVEDV* RV end-diastolic volume, *RVEF* RV ejection fraction, *RV SI* RV stroke index, *TAPSE* Tricuspid annular plane systolic excursion, *TD-CO* thermodilution-derived cardiac output, *TD-CI* TD-derived cardiac index, *TD-PVR* TD-derived pulmonary vascular resistance, *TR-Pmax* maximal tricuspid regurgitation-pressure, *TVR* tricuspid valve regurgitation, *VHD* valvular heart disease

For group comparisons, patients were divided according to the iFM_Krakau_-CO/TD-CO index into three groups: iFM_*Krakau*_ underestimated CO (index < 0.8) compared to TD in 74 (39%) patients, whereas overestimations (index > 1.2) occurred in 11 patients (5.9%). For the remaining 103 (55%) patients, the index was between 0.8 and 1.2. This group was classified as the group with “no significant deviation” (Table 2).

Patients for whom iFM_*Krakau*_ overestimated CO, i.e., ratio > 1.2, had lower TD-derived CI than patients with no significant deviation (Table 2).

By contrast, patients for whom iFM_*Krakau*_ underestimated CO, i.e., ratio < 0.8, were smaller, had a higher intake of loop-diuretics, and more often had severe aortic valve stenosis than patients with no significant deviation (Table 2). In RHC, pulmonary artery pressure, TD-CO, and TD-CI levels were higher than in patients with no significant deviation (all *P* < 0.05).

### CMR and discrepancies between iFM_Krakau_-CO and TD-CO

Reduced LV and RV stroke indexes were the only variables that showed a trend toward a deviation of > 20% between iFM_Krakau_-CO and TD-CO (*p* = 0.066 and *p* = 0.052 in patients with no deviation vs. a ratio > 1.2, respectively). All other variables were not significantly different between groups (Table 2).

### Correlations between different CO methods

**Fig. 1:**
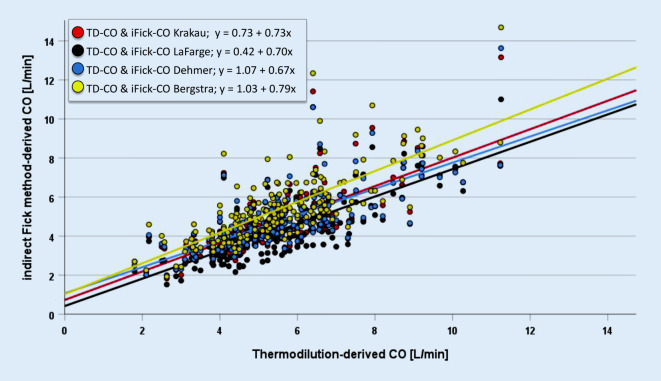
Cardiac output (*CO*) determined by thermodilution (*TD*) compared to various indirect Fick methods with best-fit lines and simple linear regression

Table 3 summarizes the differences and correlations between TD-derived CO, cardiac index (CI), and oxygen consumption ($\dot {\mathrm{V}}$O_2_, mL/min) compared to different iFM methods. Associations between CO derived by TD and iFM methods were visualized in a scatter plot, including best-fit lines for linear regression models for each equation (Fig. 1).

**Table 3:** Comparison of thermodilution-derived cardiac output, cardiac index, and oxygen consumption with different indirect Fick methods

	TD	iFM_Krakau_	iFM_LaFarge_	iFM_Dehmer_	iFM_Bergstra_
*CO [L/min]*	5.4 (4.4; 6.5)	4.4 (3.7; 5.5)	4.1 (3.2; 4.8)	4.5 (3.8; 5.5)	5.1 (4.2; 6.1)
P^TD vs. others^	–	< 0.001	< 0.001	< 0.001	0.007
R^TD vs. others^	–	0.76 (0.70; 0.82)*	0.76 (0.70; 0.82)*	0.75 (0.68; 0.80)*	0.76 (0.69; 0.81)*
*CI [L/min/m*^*2*^*]*	2.8 (2.4; 3.3)	2.3 (2.0; 2.8)	2.1 (1.7; 2.5)	2.3 (2.0; 2.8)	2.6 (2.2; 3.1)
P^TD vs. others^	–	< 0.001	0.001	< 0.001	0.002
R^TD vs. others^	–	0.73 (0.66; 0.79)*	0.71 (0.64; 0.78)*	0.72 (0.64; 0.78)*	0.72 (0.65; 0.78)*
$\dot {\mathrm{V}}$*O*_*2*_* [mL/min]*	277 (238; 324)	242 (214; 264)	223 (190; 244)	243 (223; 260)	274 (245; 298)
P^TD vs. others^	–	< 0.001	< 0.001	< 0.001	0.007
R^TD vs. others^	–	0.52 (0.41; 0.63)*	0.52 (0.41; 0.62)*	0.51 (0.39; 0.62)*	0.52 (0.41; 0.62)*

Median and quartiles of cardiac output, cardiac index, and oxygen consumption according to thermodilution and different indirect Fick methods; *p* values for differences are shown for CO, CI, and $\dot {\mathrm{V}}$O_2_ (each for TD vs. others) according to Wilcoxon rank test. Further Pearson correlation analysis (*r*; 95% confidence intervals, 95% CI) for CO, CI, and $\dot {\mathrm{V}}$O_2_ are shown (each for TD vs. others)

*CO* cardiac output, *CI* cardiac index, *VO*_*2,*_ oxygen consumption, *TD* thermodilution, *iFM* indirect Fick methods

*p < 0.001

The TD-CO was significantly higher than all iFM-derived CO (Table 3; all *p* > 0.05). The correlation coefficients between TD-CO were similar in magnitude for all iFM (*r* = 0.75–0.76; *p* > 0.001 Table 3). Associations were similar, albeit numerically smaller, when CI or $\dot {\mathrm{V}}$O_2_ instead of CO values were compared (Table 3).

When iFM_*Krakau*_-CO was compared with the other iFM-CO levels, significant differences were seen for all methods (all *p* ≤ 0.001), except for iFM_*Dehmer*_ (*p* = 0.19). Associations were similar when iFM_Krakau_-CI and iFM_Krakau_-$\dot {\mathrm{V}}$O_2_ were compared with the other iFM measures (CI: *p* ≤ 0.001, except for iFM_Dehmer_ *p* = 0.12; $\dot {\mathrm{V}}$O_2_: *p* all ≤ 0.001, except for iFM_Dehmer_ *p* = 0.15).

### Level of agreement between different CO methods

Table 4 illustrates the percentage of deviations > 20% from TD-derived CO measurements for each iFM calculated. As shown, iFM_*LaFarge*_ yielded the highest proportion of total deviations (64%), mainly via a high number of patients with a ratio < 0.8 (60%). By contrast, iFM_*Bergstra*_ showed opposing associations (28% deviations > 20%), while iFM_*Krakau*_ and iFM_*Dehmer*_ performed in-between (deviations > 20%; 45% and 43%). Concerning interrater reliability, iFM_*Dehmer*_ had the highest Cohen’s kappa coefficient among the calculated iFM estimates compared to iFM_*Krakau*_.

**Table 4:** Discrepancies greater than 20% between different iFM-CO methods and TD-CO

	iFM-CO/TD-CO			Cohen’s κ (95% CI)
	< 0.8 (*N*, %)	0.8–1.2 (*N*, %)	> 1.2 (*N*, %)	
iFM_Krakau_-CO/TD-CO	74 (39.4)	103 (54.8)	11 (5.9)	–
iFM_LaFarge_-CO/TD-CO	113 (60.1)	68 (36.2)	7 (3.7)	0.58 (0.47; 0.68)*
iFM_Dehmer_-CO/TD-CO	68 (36.2)	107 (56.9)	13 (6.9)	0.75 (0.65; 0.84)*
iFM_Bergstra_-CO/TD-CO	29 (15.4)	136 (72.3)	23 (12.2)	0.43 (0.32; 0.55)*

Cross tables showing the number of patients (*N*; %) with and without a deviation of > 20% when comparing various indirect Fick method-derived CO with thermodilution-derived CO. Interrater reliability between iFM_Krakau_-CO/TD-CO and different iFM-CO/TD-CO indexes is shown as Cohen’s kappa

*TD* thermodilution, *CO* cardiac output, *CI* cardiac index, *iFM* indirect Fick methods

*p < 0.001 for all

**Fig. 2:**
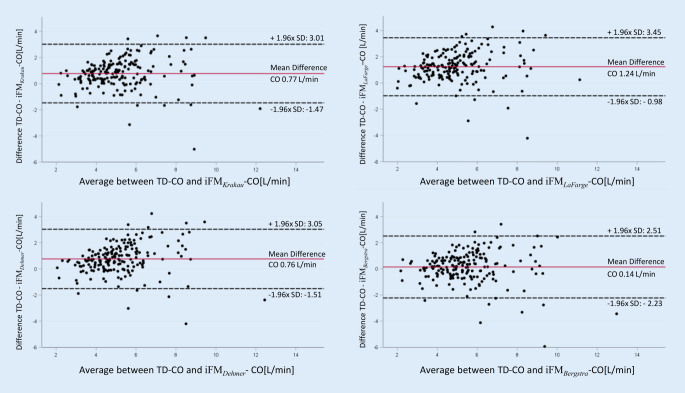
Bland–Altman plots comparing the cardiac output (*CO*) measured by thermodilution (*TD*; reference standard) and four indirect Fick (*iFM-CO*) methods

The level of agreement between TD and different iFM methods was further depicted in Bland–Altman plots (Fig. 2). The lowest mean difference (0.14 L/min), but numerically also the broadest limits of agreement (+2.51 to −2.23) were seen for iFM_*Bergstra*_, while iFM_*LaFarge*_ had the largest mean difference (1.24 L/min) and numerically the narrowest limits of agreement (3.45 to −0.98; Fig. 2). Mean differences between TD-CO and iFM_*Krakau*_ and iFM_*Dehmer*_-derived CO were 0.77 L/min and 0.76 L/min, with similar limits of agreements (iFM_Krakau_ 3.01 to* −*1.47; iFM_Dehmer_ 3.05; −1.51 Fig. 2). Percentage errors between TD-CO and different iFM were similar, but all greater than 30% (iFM_*Krakau*_/iFM_*LaFarge*_/iFM_*Dehmer*_/iFM_*Bergstra*_ 44%/45%/44%/43%), indicative of poor comparability.

When iFM_Krakau_ was compared with the other iFM, the lowest mean difference (0.01 L/min) was seen for the comparison with iFM_Dehmer_ (limits of agreement −0.65 to 0.63 L/min), but also the comparisons with iFM_LaFarge_ (mean difference 0.46 L/min; limits of agreement +1.15 to −0.22) and iFM_Bergstra_, (mean difference −0.63 L/min; limits of agreement −1.2 to −0.05) were more related than with the TD method. Percentage errors between iFM_Krakau_ and other iFM were all less than 30% (iFM_LaFarge_/iFM_Dehmer_/iFM_Bergstra_ 15%/13%/11%).

### Determinants of a significant discrepancy

Table 5 lists the baseline characteristics predicting a deviation of > 20% between iFM_Krakau_-derived and TD-derived CO in univariable logistic regression (age and sex forced into the analysis). Significant predictors were included in a multivariable model (age and sex forced into the analysis), in which male sex was associated with a reduced risk, while high-grade aortic valve stenosis and higher mean pulmonary artery pressure were associated with an increased risk of a significant deviation (Table 5)*.*

**Table 5:** Determinants of a discrepancy of > 20% between thermodilution and iFM_Krakau_-derived CO

Predictors of iFM_*Krakau*_-CO/TD-CO index < 0.8 or > 1.2	Univariable analysis OR (95% CI)	Multivariable analysis OR (95% CI); *p*
Age, per decade	1.12 (0.92; 1.37); *p* = 0.27	**–**
Male sex, yes vs. no	0.56 (0.30; 1.05); *p* = 0.070	0.50 (0.26; 0.98); ***p*** **=** **0.044**
Body height, per cm	0.96 (0.93; 0.99); ***p*** **=** **0.022**	**–**
AVS grade °III, yes vs. no	4.54 (1.59; 13.0); ***p*** ***=*** **0.005**	4.21 (1.43; 12.0); ***p*** **=** **0.011**
Loop diuretics, yes vs. no	1.85 (1.00; 3.42); ***p*** **=** **0.049**	**–**
Mean PAP, per mm Hg	1.04 (1.01; 1.07); ***p*** **=** **0.006**	1.04 (1.01; 1.07); ***p*** **=** **0.007**

Univariate and multivariate logistic regression in patients with iFM_Krakau_-CO/TD-CO < 0.8 or > 1.2 as the dependent variable. Independent predictors are highlighted in bold; age and sex were forced into the analysis

*AVS* aortic valve stenosis, *OR* odds ratio, *PAP* pulmonary artery pressure

## Discussion

In this retrospective cohort study, we investigated the agreement between TD-derived CO and CO derived from four different equations for $\dot {\mathrm{V}}$O_2_ estimation, including a non-validated formula proposed by Krakau [17]. We further determined independent predictors of a significant deviation between the measured and the estimated method, according to Krakau.

The principal findings were:

1. None of the formulas tested showed good agreement with the reference standard, but the Krakau formula correlated highly with the Dehmer equation in different method-comparison analyses.
2. None of the variables assessed in routine CMR were predictive of an increased risk for a discrepancy of > 20% between iFM_Krakau_ and TD-derived CO, but female sex, high-grade aortic valve stenosis, and higher pulmonary pressure were independent predictors in multivariable logistic regression.

Right heart catheterization is indicated whenever relevant diagnostic information or therapeutic consequences are expected from the results [25]. In the last few years, the number of RHC procedures has been continuously decreasing, as the procedure was primarily recommended for evaluation of pulmonary hypertension, advanced heart failure, and specific conditions such as congenital heart diseases, pericarditis, and restrictive cardiomyopathies [1, 26]. However, RHC may also provide important prognostic and diagnostic information in valvular heart disease (VHD) and (non-advanced) HFrEF, especially in those with discordant clinical findings [25]. Further, RHC is demanded for basic cardiology training, thus, regular examinations are mandatory in training centers to address educational requirements. One of the critical measures in RHC is the accurate assessment of CO.

The direct Fick and the TD method are guideline-advocated methods for measuring CO. As the direct Fick method is not suitable for routine application in the cardiac catheter laboratory for various reasons, TD is regarded as the clinical gold standard despite the multiple caveats associated with this method [27].

In clinical practice, the iFM may be preferred over the gold standards for feasibility reasons. However, there are multiple $\dot {\mathrm{V}}$O_2_ estimation formulas with significant in-between differences [11, 13, 28, 29]. Although these variations are widely published, even renowned journals do not request citing the exact equations used for CO estimation [15, 16]. The largest study comparing TD-derived CO with iFM-derived CO analyzed 12,232 patients from the Veterans Cohort and 3197 patients from the Vanderbilt cohort and showed that TD-derived CO was more effective in predicting mortality than iFM-derived CO but without specifying the formulas used for $\dot {\mathrm{V}}$O_2_ estimation [15]. Fares et al. studied a cohort of patients with pulmonary hypertension and found a difference of > 20% between TD-CO and iFM-CO in 36% of patients but did not reference the formula(s) used for $\dot {\mathrm{V}}$O_2_ estimation [16]. The Krakau formula [17], by contrast, is mentioned as a standard for $\dot {\mathrm{V}}$O_2_ estimation in various cardiology textbooks [17, 30, 31] and publications[32, 33] although its study was never validated and no information on its derivation cohort has been published to date. We showed that iFM_Krakau_-derived CO did not agree with the gold standard TD-CO, but the associations were not worse than with the scientifically better evaluated approximation formulas proposed by LaFarge and Miettinen [8], Dehmer et al. [9], and Bergstra et al. [10]. In fact, the Krakau formula agreed in all method-comparison analyses (percentage errors, median levels, mean differences, levels of agreement) reasonably well with the formula proposed by Dehmer et al. [9]. This was also the only study that did not include children in the derivation cohort (*N* = 164; [9]). Here, the mean age of the adult patients was 50 years (age range: 21–75 years), and cardiac output was measured with either the TD (*N* = 89) or the dye dilution (*N* = 75) method. The cohorts from which the other formulas originate are not comparable with the standard adult patients receiving RHC today [11].

LaFarge and Miettinen investigated a predominantly pediatric cohort (76% aged 3–16 years) to derive the formula, using age, BSA, and heart rate as determinants of measured $\dot {\mathrm{V}}$O_2_ levels [8]. In most of the studies described, including the current one, this formula tended to underestimate $\dot {\mathrm{V}}$O_2_ and consequently the CO levels, and regularly yielded the highest number of patients with significant discrepancies from TD-CO [11, 13, 29].

The formula by Bergstra et al. [10] that originates from a cohort of 250 patients with a mean age of 34.6 ± 22 years (range: 1–84 years), in which $\dot {\mathrm{V}}$O_2_ levels were estimated by the indicator-dilution method, regularly overestimates $\dot {\mathrm{V}}$O_2_ and CO levels [11, 13, 28]. Patients were generally poorly characterized in all the aforementioned derivation cohorts. There are a couple of characteristics that may affect the results of iFM-CO or TD-CO assessment and thus alter the congruity between both methods.

Higher-grade tricuspid regurgitation (TR) and heart failure are alleged to be critical confounders of TD-CO assessment and hence may explain a higher degree of discrepancy between TD- and iFM-derived CO [5]. In our study, *neither *reduced ejection fraction *nor* high-grade TR were predictive of a CO discrepancy > 20%.

There are conflicting data on whether the TD procedure is methodologically suitable in the presence of higher-grade TR, which is why some authors in this situation endorse the Fick (or iFM) method [5, 34, 35]. As there may be backflow of a certain amount of the injected volume, a lower amount of the predefined cold volume reaches the tip of the catheter in time, which subsequently may lead to a reduction or “underestimation” of CO by the TD method in high-grade TR [34, 35]. Other clinical or experimental studies could not confirm a significant discrepancy between TD-derived and Fick-derived CO [36–38].

We hypothesized that reduced right ventricular (RV) function may have confounded previously described associations of high-grade TR and reliability of CO measurement by the TD method. But neither echocardiographic (tricuspid annular plane systolic excursion, RV end-diastolic diameter) nor CMR-derived parameters of RV function and morphology (RV ejection fraction, RV end-diastolic volume, RV-CO, RV stroke index) were significantly associated with a higher number of discrepancies between iFM-derived and TD-derived CO in our study.

In our cohort, female sex, higher pulmonary artery pressure, and high-grade aortic valve stenosis were independent predictors of an increased risk for discrepancies. While female sex and pulmonary hypertension are well-known predictors of relevant differences between the iFM and the TD method, the associations for aortic valve stenosis are less clear [11, 16].

The lower agreement between TD-derived and iFM-derived CO in patients with higher-grade AS was already shown in another study, in which the correlation coefficient between both methods was only *r* = 0.56 [39]. Associations were similar in our study when patients with high-grade AS were selected (according to different iFM methods: *r* = 0.52–0.59), but worse than in the total cohort (according to different iFM methods: *r* = 0.74–0.76).

The reason for the worse correlation in AS is unclear, but demonstrates once again that there is no “one-fits-all” $\dot {\mathrm{V}}$O_2_ approximation model that can be used for every patient. Since the characteristics of patients receiving RHC today are significantly different than in the period 1970–1999, when the respective $\dot {\mathrm{V}}$O_2_ approximation formulas were published[8–10, 17], the results are not surprising, but demonstrate once again, that currently the best methods for CO measurement are the established ones advocated by the guidelines [1].

### Limitations

Our single-center study has some limitations, such as the retrospective design, the nonstandardized mode of data collection, and the exclusion of one third of the patients identified due to missing values. Additionally, as availability of CMR was an inclusion criterion for this study, the cohort was younger and healthier than the average patient presenting with RHC. Thus, the study did not include patients with non-CMR-compatible implantable devices, severe dyspnea when lying flat, or severe renal dysfunction. However, proper characterization of the cardiac chambers in CMR was one of the primary objectives of this study and the results of comparative analyses between different iFM-CO and TD-CO were similar to already published data. Further, we did not measure CO according to the direct Fick method. Since TD is regarded as the clinical gold standard and direct $\dot {\mathrm{V}}$O_2_ measurements are not performed routinely in most catheter laboratories anyway, results still reflect real-world conditions.

## Conclusion

In conclusion, the as-yet scientifically unfathomed $\dot {\mathrm{V}}$O_2_ estimation formula proposed by Krakau performed better than the formula introduced by LaFarge and Miettinen, worse than the formula of Bergstra et al., and comparable to the formula proposed by Dehmer at al., when iFM-derived CO was calculated and the number of significant discrepancies from TD-derived CO was compared. However, none of the formulas showed good agreement with TD-derived CO. Newer derivation cohorts and formulas are needed for the estimation of $\dot {\mathrm{V}}$O_2_ that are more comparable with today’s patients presenting with RHC.

## Declarations

All procedures performed in studies involving human participants or on human tissue were in accordance with the ethical standards of the institutional and/or national research committee and with the 1975 Helsinki declaration and its later amendments or comparable ethical standards. Approval of the ethical committee was waived as the Data Ware House runs on standard operating procedures that are controlled and approved by the institution’s data protection officer. Informed consent was waived due to the retrospective nature of the study and anonymous data analysis.
